# Promoting intervertebral disc fusion using BMP2, L51P, and the EP4 agonist KMN-159: synergistic osteogenesis in human annulus fibrosus cells

**DOI:** 10.1007/s00586-026-09841-0

**Published:** 2026-03-03

**Authors:** Shuimu Chen, Xinggui Tian, Stefan Zwingenberger, Christoph E. Albers, Benjamin Gantenbein, Sonja Häckel

**Affiliations:** 1 Department of Orthopaedic Surgery & Traumatology, Inselspital, Bern University Hospital, Faculty of Medicine, University of Bern, CH-3010 Bern, Switzerland; 2 Tissue Engineering for Orthopaedics & Mechanobiology (TOM), Bone & Joint Program, Department for Bio-Medical Research (DBMR), Faculty of Medicine, University of Bern, CH-3008 Bern, Switzerland; 3 Graduate School for Cellular and Biomedical Sciences (GCB), University of Bern, CH-3012 Bern, Switzerland; 4 Center for Orthopaedic, Trauma Surgery and Rehabilitation Medicine, University Medicine Greifswald, 17475 Greifswald, Germany; 5 Orthopäädie Sonnenhof, KLG, CH-3006 Bern, Switzerland; 6 Graduate School for Health Sciences(GHS), University of Bern, CH-3012 Bern, Switzerland

**Keywords:** Spinal fusion, Annulus fibrosus cells, KMN159, BMP2, L51P, Osteogenesis

## Abstract

**Purpose:**

Spinal fusion surgery is widely used to restore spinal stability but remains challenging in elderly patients with comorbidities. This study aimed to explore whether combining bone morphogenetic protein-2 (BMP2), its analogue L51P, and a prostaglandin E2 receptor 4 agonist KMN159 could induce osteogenic differentiation of human annulus fibrosus cells (AFCs), thereby providing a new biological strategy for spinal fusion.

**Methods:**

Primary human AFCs (*n* = 11) were cultured under osteogenic conditions and treated with BMP2, L51P, and KMN159 individually or in combination. Cell viability was evaluated using the tetrazolium salt (XTT) assay, osteogenic differentiation was assessed by alkaline phosphatase (ALP) activity, bone-related gene expression (ALP, SP7, COL1), and calcium deposition by alizarin red staining. Expression of BMP antagonists (Noggin, Gremlin1, Chordin) was also quantified by qPCR.

**Results:**

KMN159 alone showed no cytotoxicity but weaker osteogenic induction than BMP2. When combined with BMP2 and L51P, KMN159 significantly enhanced ALP activity, bone-related gene expression trend, and calcium deposition, with the triple combination showing the strongest effect. Synergistic osteogenesis was particularly evident in AFCs from elderly patients, females, degeneration-derived discs, and lower lumbar segments.

**Conclusion:**

The combination of BMP2, L51P, and KMN159 effectively promotes osteogenic differentiation of human AFCs in vitro. This minimally invasive strategy may enable disc-preserving spinal fusion, offering potential therapeutic benefits for patients with degenerative disc disease and low back pain.

## Introduction

The body’s response to advanced intervertebral disc (IVD) degeneration is a natural fusion with osteophyte formation, and proper bone formation (ossification) is typically observed at the margins of the disc, especially where the disc interfaces with the vertebral body via the cartilage endplate. Moreover, 63% of the elderly population present with calcification in the annulus fibrosus (AF) [1], which makes these cells an excellent target for inducing bone formation. Unfortunately, this “auto fusion” is not always successful, and patients still experience micro instability due to degenerative changes, including facet joint arthrosis and a vacuum phenomenon in the disc [2]. This often requires surgical treatment in the form of spinal fusion, which remains the gold standard for treatment of disc degeneration. However, complications such as pseudarthrosis, cage subsidence, and implant-related infections continue to pose significant problems [3].

Biological approaches that further enhance osteogenesis and fusion rates while minimizing complications represent a paradigm shift toward safer and more effective biologic spinal fusion [4–6]. Therefore, biological augmentation has emerged, incorporating agents like bone morphogenetic protein 2 (BMP2), which possesses strong osteoinductive properties and promotes bone formation [7, 8]. Nevertheless, the clinical application of BMP2 has been limited by high costs [8] and inconsistent outcomes due to natural BMP antagonists in the IVD, such as Noggin, Gremlin 1, and Chordin [9, 10]. The use of BMP2 analogues, such as L51P (a Leucine-51-to-Proline mutant), reduces the inhibitory effects of BMP2 antagonists [6]. A further study indicates that L51P can enhance BMP2-induced osteogenic differentiation at the same dose and that this effect becomes progressively stronger as the L51P dose increases [11]. It is well known that BMP2 has side effects like ectopic bone formation, inflammation, which could lead to osteolysis, nerve damage, and wound problems. However, doses as low as 0.5–1.0 mg/level in lumbar fusion have been shown to balance efficacy and safety, minimizing complications without compromising fusion rates [12].

Recently, KMN159, a prostaglandin E2 receptor 4 (EP4) agonist developed by Cayman Chemical, Inc. (Ann Arbor, MI, USA), has demonstrated potential to promote bone formation and osteogenic differentiation both in vitro and in preclinical animal models. KMN159 was tested in unfractionated rat bone marrow cells (BMCs), osteoblasts, osteoclasts, and in vivo in rat models of bone defect and spinal fusion. In vitro, KMN159 induced robust osteoblastic differentiation, as evidenced by increased alkaline phosphatase, type I collagen, and osteocalcin expression. It also promoted osteoclastogenesis in the presence of RANKL but did not increase adipogenesis. In vivo, local delivery of KMN159 via mineralized collagen matrices resulted in dose-dependent new bone formation, improved vascularization, and enhanced biomechanical properties of the fusion mass [4, 5, 13].

Given the complementary functional properties of L51P and KMN159, their combination with BMP2 may produce additive or synergistic osteoinductive effects, thereby enabling a reduction in BMP2 dose while maintaining or enhancing fusion efficacy. Notably, the receptor EP4 is expressed in IVD cells [14]. Accordingly, this study investigates a minimally invasive biological strategy that combines BMP2, L51P, and KMN159 to induce osteogenic differentiation in IVD cells, specifically AFCs, with the goal of promoting spinal fusion without complete excision of degenerated disc tissue.

## Materials and methods

### Study design

**Fig. 1:**
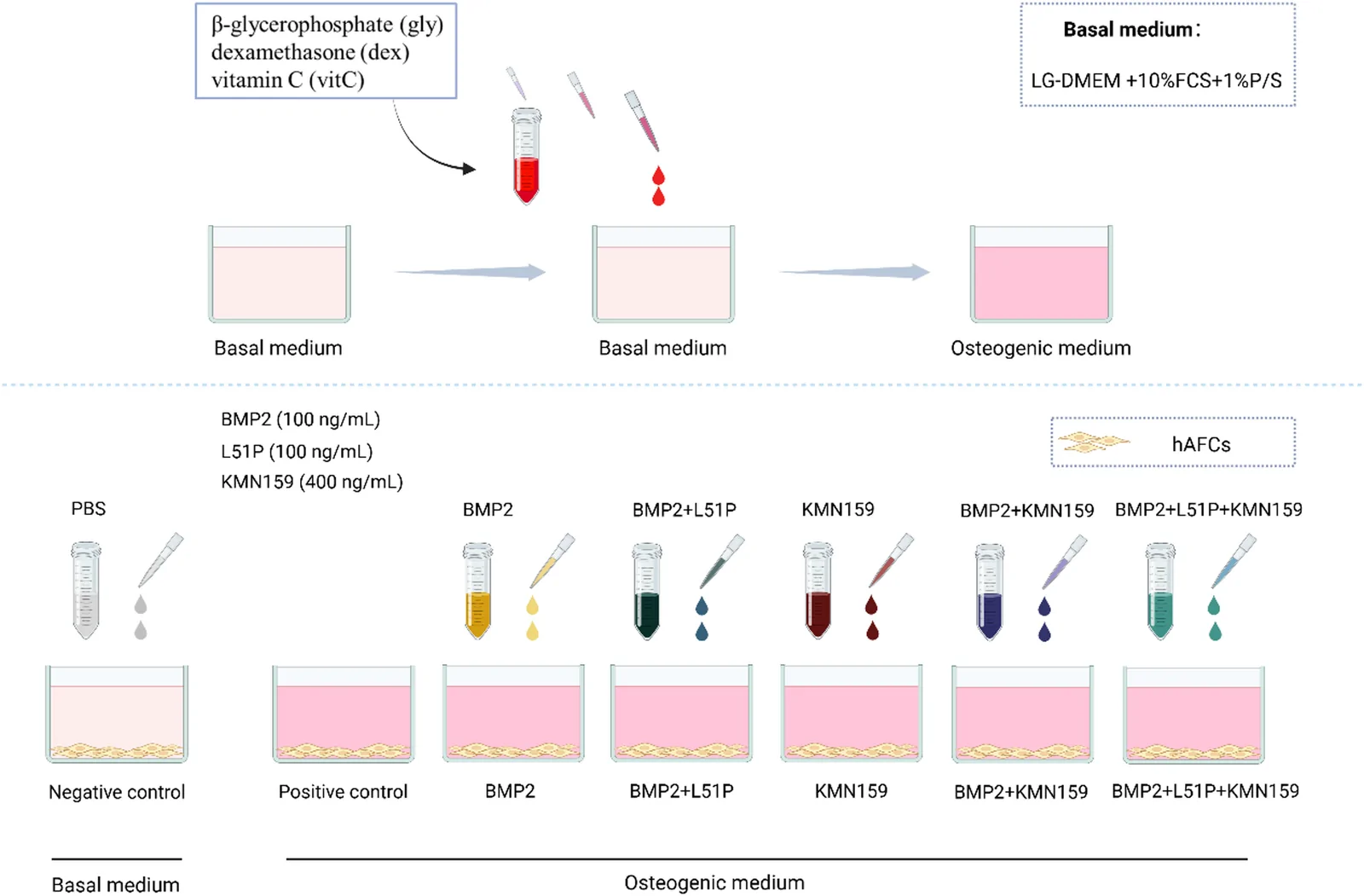
Experimental design for the osteogenic induction of human AFCs. AFCs were divided into seven groups and cultured in either basal medium (negative control) or osteogenic medium supplemented with BMP2 (100 ng/mL), L51P (100 ng/mL), and/or KMN159 (400 ng/mL), as indicated

Human AFCs were cultured to evaluate osteogenic potential under different bioactive factor or their combinations. Cells were maintained in basal medium [low-glucose (1 g/L) DMEM with 10% FBS and 1% penicillin/streptomycin] or in osteogenic medium (basal medium supplemented with 0.1 mM L-ascorbic acid-2-phosphate, 20 mM β-glycerophosphate, 200 nM dexamethasone). Seven experimental groups were studied, and fresh factors were added at each medium change (Fig. 1). The concentrations of BMP2, L51P, and KMN159 were determined based on previously published studies [5, 15].

### Human materials and cell isolation

Intervertebral disc tissues were obtained from patients undergoing spine surgery at the Insel University Hospital Bern (*n* = 11). The Swiss Human Research Act does not apply to research involving anonymized biological material and/or anonymized health-related data. Therefore, this project did not require ethics committee approval. General Consent which also covers anonymization of health-related data and biological material was obtained. The AF tissue was dissected from IVD specimens by an experienced spine surgeon, washed with PBS, and enzymatically digested with pronase (1.9 mg/ml, 1 h) followed by collagenase II (128 U/ml, overnight, at 37 °C). The cell suspension was filtered (100 μm), and the resulting AFCs were expanded in low-glucose DMEM supplemented with 10% FBS and 1% penicillin/streptomycin before experimental use (Table 1).

**Table 1:** Human IVD tissues were collected to isolate AFCs in this study

Donor	Age	Gender	Segment	Degeneration	Trauma
1	28	M	L1-L2	-	√
2	30	M	L1-L2	-	√
3	28	M	T12-L1	-	√
4	55	F	L2-L3	-	√
5	30	M	L5-S1	√	-
6	25	F	L5-S1	√	-
7	64	F	L3-L4	-	√
8	60	F	L3-L4	√	-
9	78	M	L5-S1	√	-
10	72	M	L3-L4	√	-
11	66	F	L1-L2	√	-

Donor information for AFCs after MRI. M, male; F, female; T, thoracic; L, lumbar; S, sacral; √, collected; -, no data. AFCs were at passage 1–4 for the experiment

### Cell viability assay

AFCs (2,000 cells/well, 96-well plates) were cultured up to 14 days. Cell viability was assessed using a water-soluble yellow tetrazolium salt (XTT assay (R&D Systems) according to the manufacturer’s instructions, with absorbance read at 450 nm.

### Alkaline phosphatase (ALP) activity

AFCs (30,000 cells/well, 12-well plates) were cultured for 14 days under experimental conditions. Cells were lysed, and ALP activity was quantified with a phosphatase assay kit (Sigma-Aldrich) by measuring absorbance at 405 nm.

### RNA extraction and relative gene expression by quantitative PCR (qPCR)

RNA was extracted from AFCs (Day 21, 50,000 cells/well) and reverse-transcribed to cDNA. qPCR was performed (Bio-Rad CFX96) using SYBR Green and primers for osteogenic markers (ALP, SP7, COL1) and BMP antagonists (Noggin, Chordin, Gremlin1), with 18S ribosomal RNA gene that served as reference gene. Relative expression was calculated with the 2 − ΔΔCt method [16]. Primer sequences are listed in Table 2. The efficiency and specificity of all primers were validated.

**Table 2:** Human gene primers for qPCR

Gene	Accession No.	Forward sequence	Reverse sequence
*18 S*	NR_145820.1	CGA TGC GGC GG C GTT ATT C	TCT GTC AAT CCT G TC CGT GTC C
*ALP*	NM_001177520.3	GTA TGA GAG TG A CGA GAA	AAT AGG TAG TCC ACA TTG T
*SP7*	NM_001173467.3	CAG GCT ATG CT A ATG ATT ACC	GGC AGA CAG TCA GAA GAG
*COL1*	NM_000089.3	GTG GCA GTG AT G GAA GTG	CAC CAG TAA GGC CGT TTG
*NOG*	NM_001078309.1	CAG CAC TAT CT C CAC ATC CG	CAG CAG CGT CTC GTT CAG
*GREM1*	NM_001191322.1	GAG AAG ACG AC G AGA GTA AGG AA	CCA ACC AGT AGC AGA TGA ACA G
*CHRD*	XM_017007394.1	GCC TCC GCT TCT CTA TCT	AAC AGG ACA CTG CCA TTG

*18 S*, reference gene 18 S; *ALP*, alkaline phosphatase; *SP7*, osterix; *COL1*, collagen type I; *GREM1*, gremlin 1; *NOG*, noggin; *CHRD*, chordin

### Histology

Mineralized matrix deposition was assessed after 21 days by alizarin red (2%) staining. Following fixation in 4% formaldehyde, stained wells were imaged and then incubated with cetylpyridinium chloride (10% in 10 mM NaPO₄) to extract bound dye. Absorbance was measured at 570 nm for quantification.

### Statistics

Comparisons between two groups were made with Student’s t-test, and among multiple groups by one-way or two-way ANOVA (GraphPad Prism 11.0.0). A p-value < 0.05 was considered statistically significant. All experiments were performed in triplicate. To account for repeated measures and donor-related variability, linear mixed models (SPSS v28) were used, including treatment as a within-subject factor and donor as a random effect. Fixed covariates included age, sex, pathology (trauma vs. degeneration), spinal level, and passage. Pairwise comparisons were Bonferroni-adjusted.

## Results

### Effect of KMN159 on cell viability

**Fig. 2:**
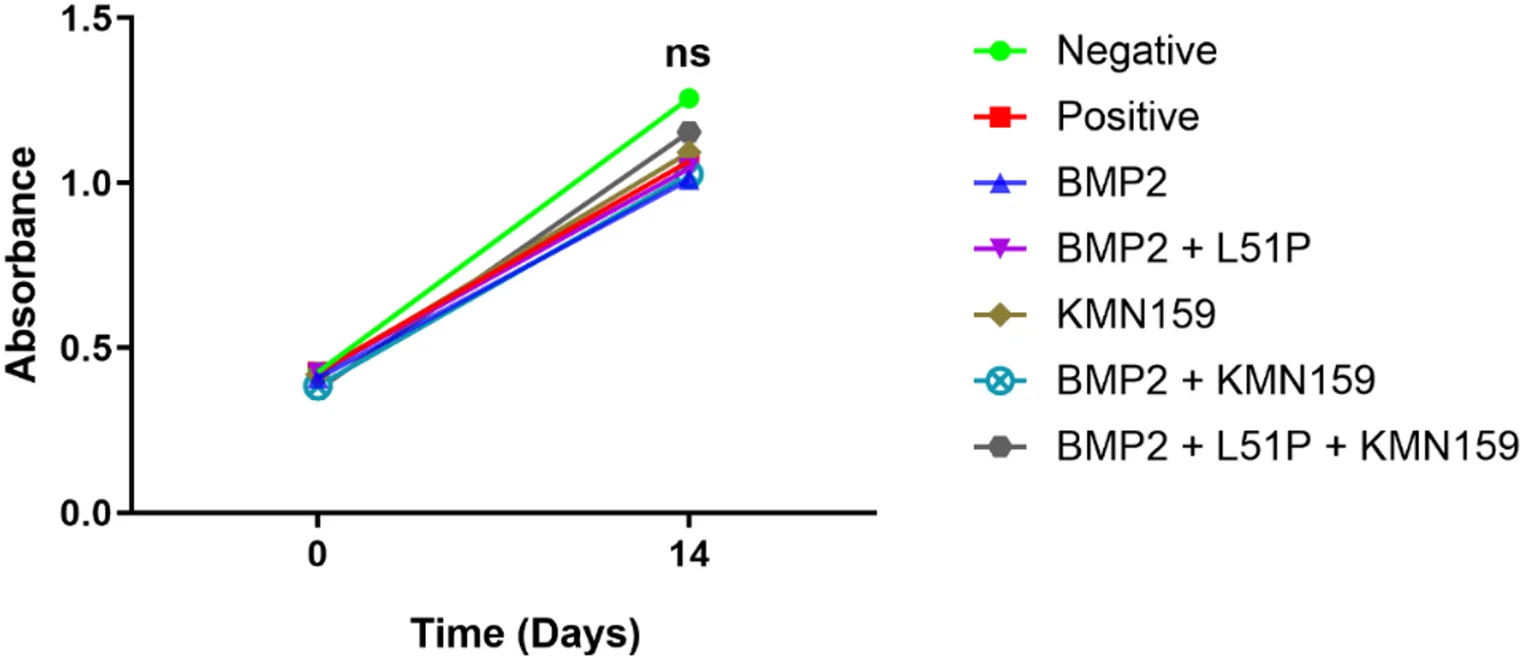
Cell viability of human AFCs assessed by XTT assay after 14 days under different treatment conditions. All groups showed increased viability by Day 14 compared to Day 0. However, no significant differences were observed among the groups (Shown as mean, p-value, ns > 0.05; *n* = 4)

To assess cytotoxicity, AFCs were cultured with KMN159 alone or in combination with BMP2 and/or L51P. XTT assays showed increased metabolic activity in all groups compared to Day 0. However, there was no significant reduction in viability across groups at Day 14 (Fig. 2). These findings indicate that KMN159 does not impair AFCs’ proliferation under the tested conditions.

### KMN159 combination with BMP2 and L51P, markedly promoted ALP activity of human AFCs

**Fig. 3:**
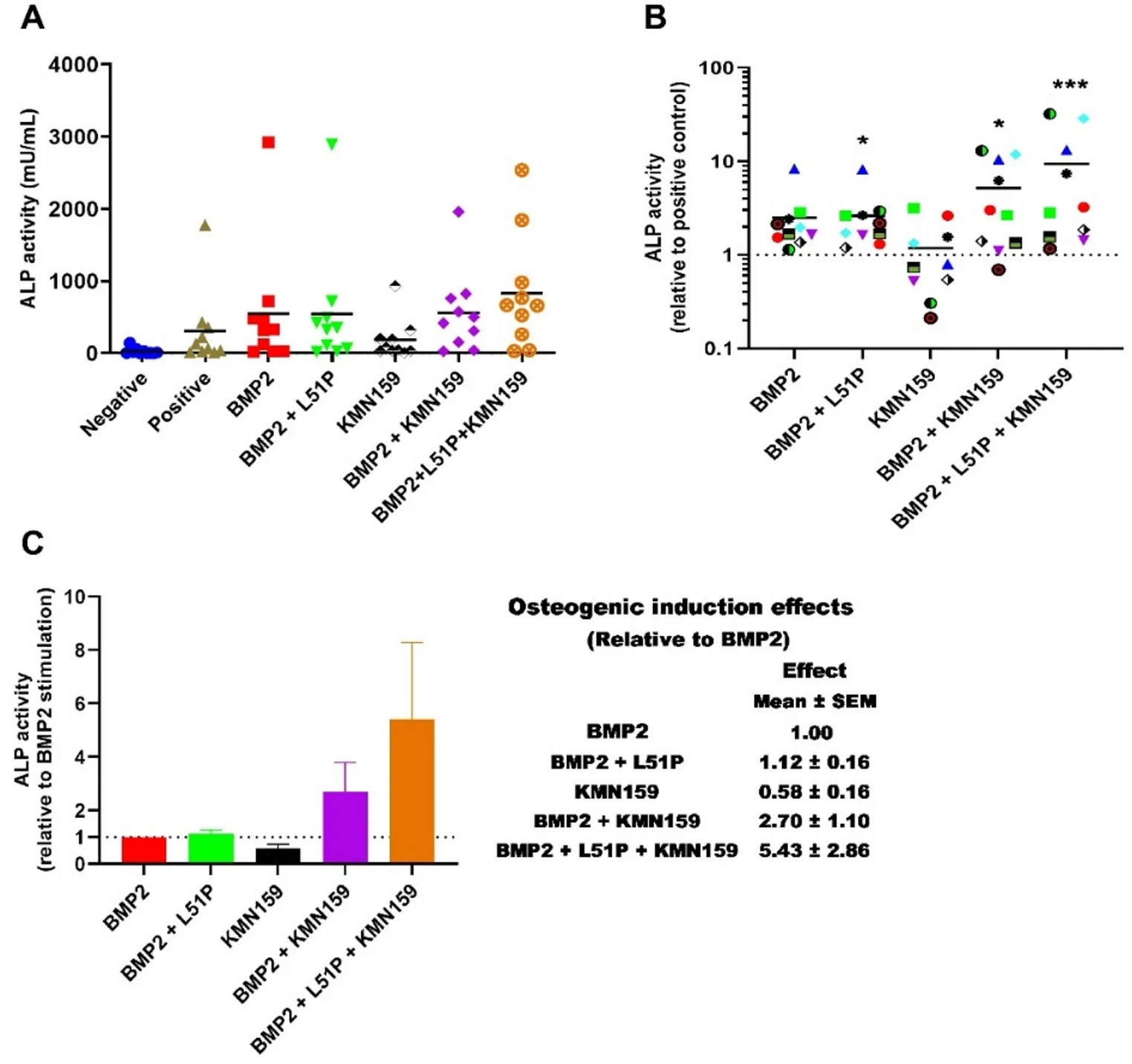
KMN159 combined with BMP2 and L51P significantly enhanced ALP activity in human AFCs. (**A**) ALP activity in AFCs after osteogenic induction under various treatment conditions. (**B**) ALP activity normalized to the positive control (osteogenic medium only). (**C**) Comparison of average ALP activity levels relative to BMP2 (set as 1.00). Data are presented as mean (mean ± SEM for **C**), p-value, * < 0.05, * * * < 0.001; *n* = 10

KMN159 alone induced only modest ALP activity compared with BMP2. In contrast, co-treatment with BMP2 or BMP2 + L51P significantly enhanced ALP activity relative to the positive control (*p* < 0.05 and *p* < 0.001, respectively). The triple combination yielded the strongest induction, with a > 5-fold increase over BMP2 alone. Relative quantitative analysis confirmed this effect, showing the highest ALP levels in BMP2 + L51P + KMN159, compared to 2.7-fold in BMP2 + KMN159 and 1.1-fold in BMP2 + L51P (Fig. 3).

### Gene expression of bone hall markers was most strongly upregulated by the triple combination (BMP2 + L51P+KMN159)

**Fig. 4:**
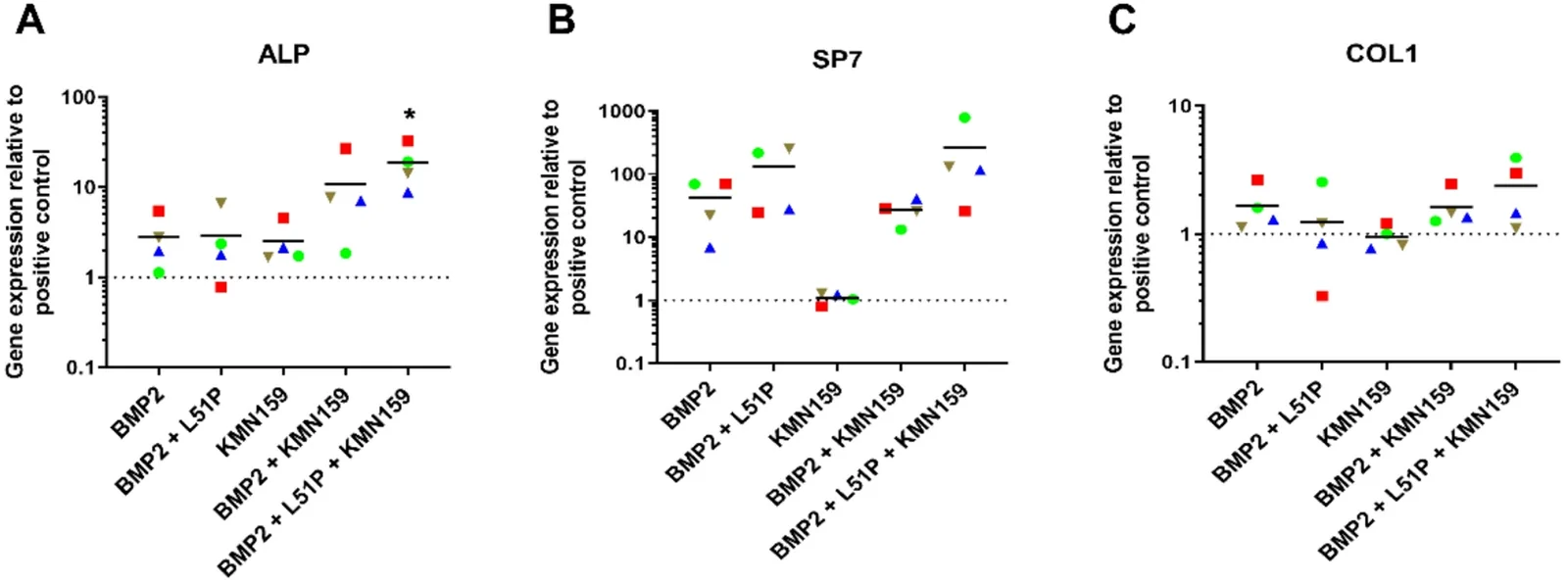
qPCR analysis of bone-related gene expression in human AFCs. (**A**) BMP2 + L51P + KMN159 significantly upregulated ALP gene expression compared to the positive group. (**B**), (**C**) An increased expression trend of SP7 and COL1 was observed in the BMP2 + L51P + KMN159 group. Data are presented as mean, p-value, * < 0.05; *n* = 4

qPCR analysis at Day 21 demonstrated that ALP mRNA expression was significantly higher in the BMP2 + L51P + KMN159 group than in the positive control (*p* < 0.05). Consistently, SP7 expression was most elevated in this group, exhibiting the greatest variability and the largest fold change across all experimental conditions. However, this effect was non-significant. Moreover, COL1 expression exhibited a steady upward trend, peaking in the triple treatment group. In contrast, KMN159 alone induced only minor alterations in the expression levels of all three genes (Fig. 4).

### KMN159 did not upregulate BMP2 antagonists

**Fig. 5:**
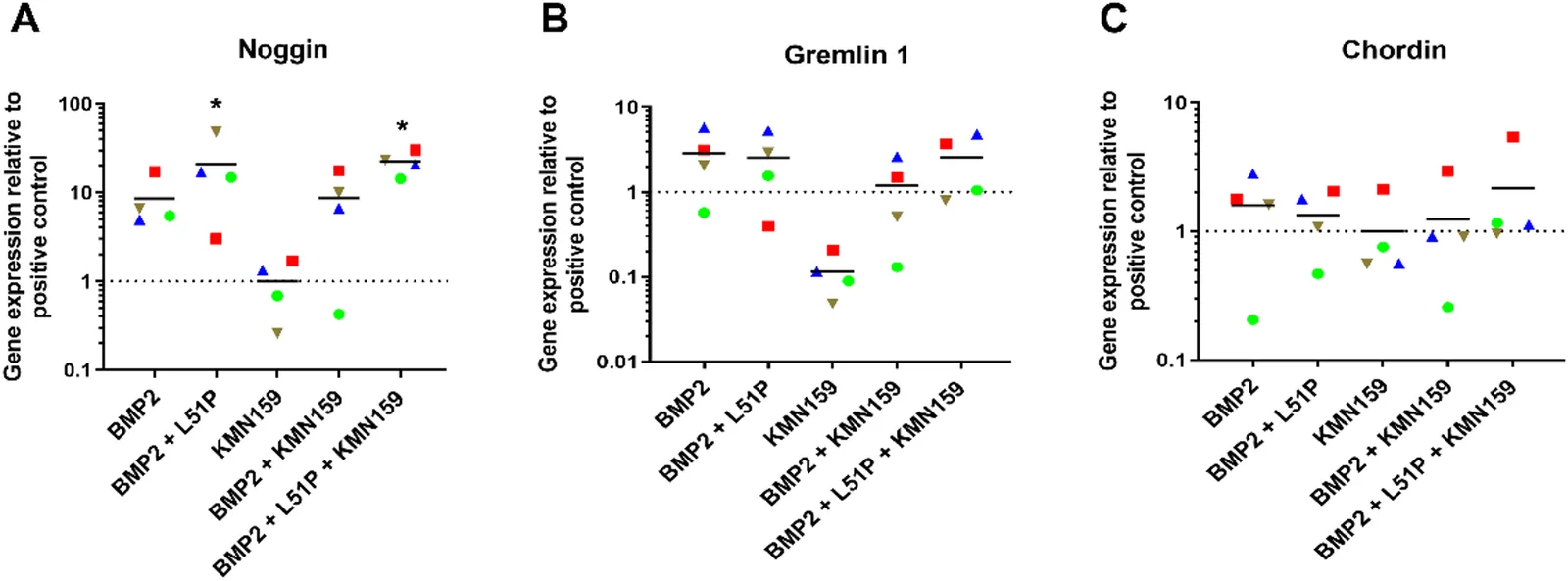
Gene expression of BMP2 antagonists in AFCs following different treatments. (**A**–**C**) mRNA levels of Noggin (**A**), Gremlin 1 (**B**), and Chordin (**C**) were quantified relative to the positive group. KMN159 alone did not upregulate BMP2 antagonists and led to minimal or even reduced expression (mean: Noggin, 1.000-fold; Gremlin 1, 0.115-fold; Chordin, 1.002-fold). Data are shown as mean, p-value, * < 0.05; *n* = 4

To evaluate whether KMN159 modulates BMP2 antagonists, qPCR was performed on Day 21 for Noggin, Gremlin1, and Chordin. KMN159 alone resulted in minimal changes or downregulation when expressed as fold-change relative to the positive control group (Noggin 1.000-fold; Gremlin1 0.115-fold; Chordin 1.002-fold). In contrast, BMP2-containing groups showed a trend of increased expression. The BMP2 + L51P + KMN159 group exhibited the highest fold-increase (Noggin 22.21-fold, Gremlin1 2.581-fold, Chordin 2.166-fold; *p* < 0.05 for Noggin). The BMP2 group showed Noggin 8.577-fold, Gremlin1 2.851-fold, Chordin 1.602-fold, while BMP2 + L51P showed Noggin 20.72-fold, Gremlin1 2.517-fold, Chordin 1.344-fold relative to positive control (Fig. 5).

### BMP2 + L51P + KMN159 strongly enhanced mineralization

**Fig. 6:**
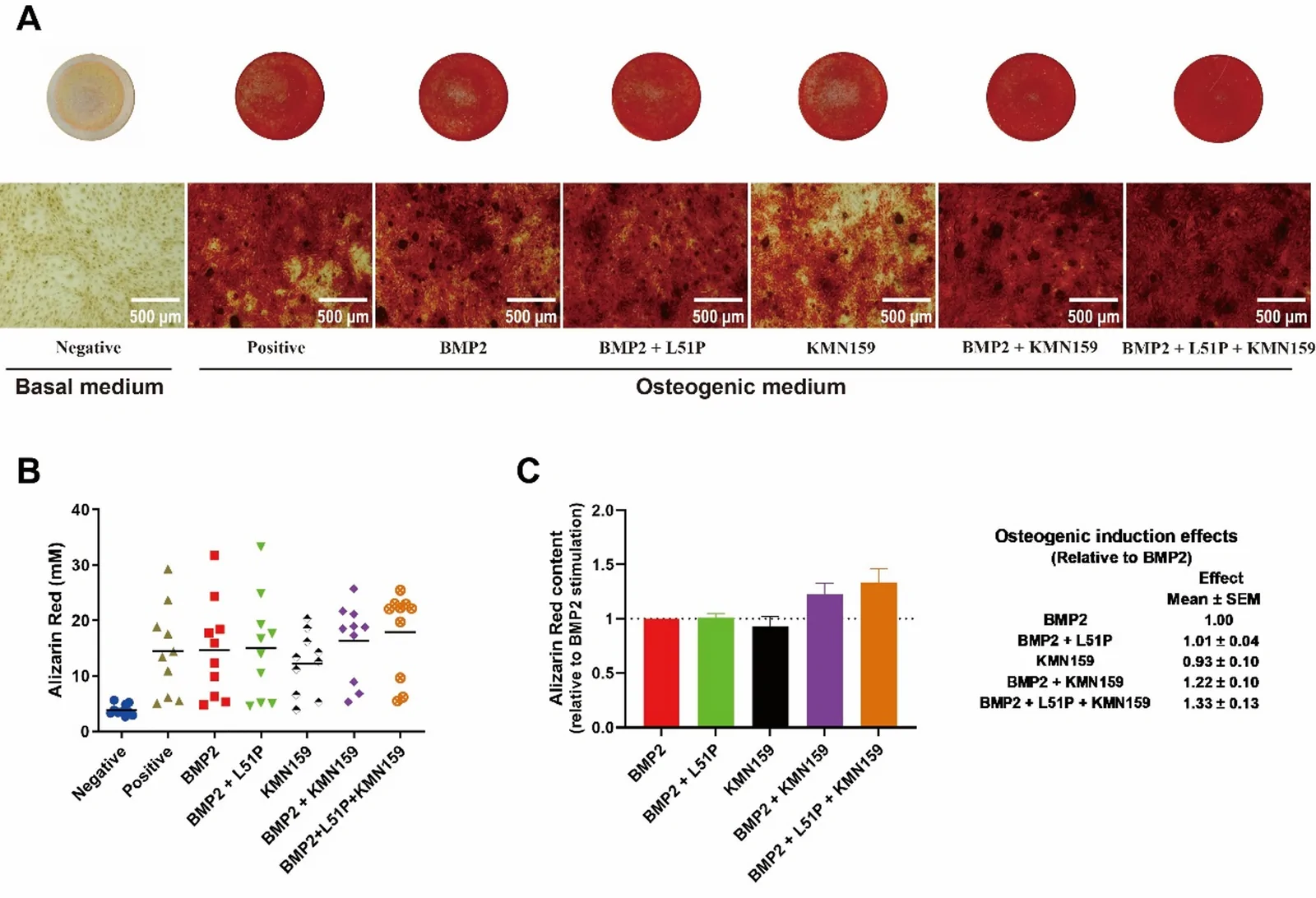
Alizarin red staining and quantification of calcium deposition in AFCs after 21 days of osteogenic induction. (**A**) Representative staining images showing that the BMP2 + L51P + KMN159 group exhibited the most intense mineralization. (**B**) Quantification of alizarin red content (mM) in different groups. (**C**) Relative mineralization levels normalized to the BMP2 group, and the BMP2 + L51P + KMN159 group, showing the highest calcium deposition, reaching a 1.33-fold increase. Data are shown as mean (mean ± SEM for **C**); *n* = 10

Alizarin red staining after 21 days demonstrated mineralized nodules in all osteogenic groups (Fig. 6A). The BMP2 + L51P + KMN159 group showed the most intense and uniform staining. Quantitative analysis confirmed the highest calcium deposition in the triple group (17.9 mM), exceeding BMP2 (14.7 mM), BMP2 + KMN159 (16.3 mM), and BMP2 + L51P (15.1 mM). Relative to BMP2 alone, mineralization was 1.33-fold higher with the triple treatment (Fig. 6B–C). KMN159 alone achieved ~ 93% of BMP2 levels, while the negative control showed minimal mineralization (3.9 mM). However, these effects were not statistically significant.

### Patient- and tissue-specific factors influenced osteogenic potential

**Fig. 7:**
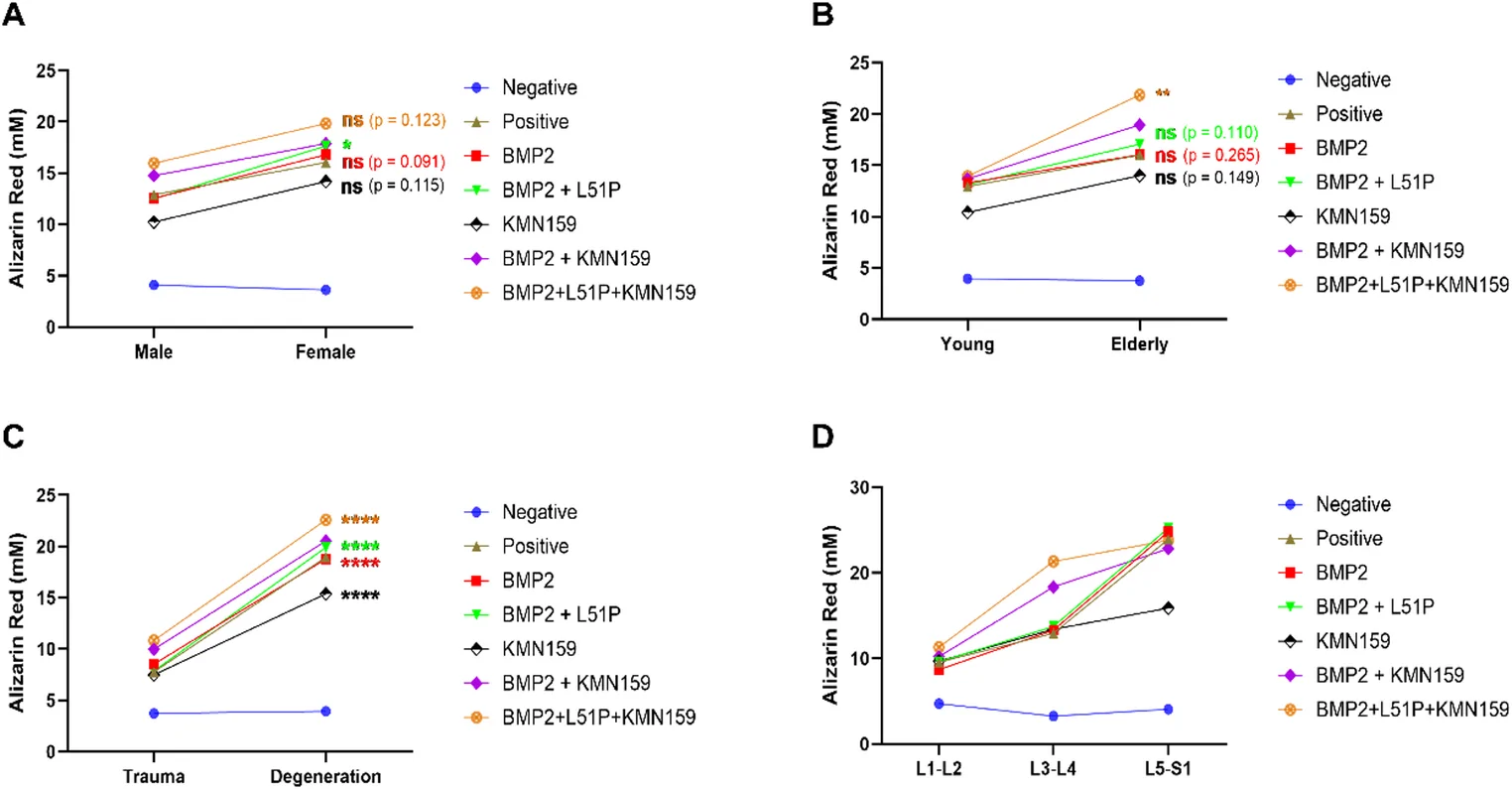
Subgroup analysis of calcium deposition in AFCs under different biological and anatomical conditions. (**A**) Female-derived AFCs exhibited greater calcium deposition than male-derived cells across most treatments. (**B**) Age-based comparison indicated increased osteogenesis in elderly (≥ 60 years) compared to younger donors. (**C**) Degeneration-derived AFCs demonstrated significantly higher mineralization than trauma-derived cells across all groups. (**D**) IVD segment analysis revealed increased calcium deposition in lower lumbar discs (L5–S1) compared to upper segments (L1–L2). Data are presented as mean; p-value, ns > 0.05; * < 0.05;* * < 0.01; * * * * < 0.0001; *n* = 10 (**A**–**C**), *n* = 9 (**D**). Donor distribution: A, male: female = 5:5; B, young: elderly = 5:5; C, trauma: degeneration = 4:6; D, spinal levels L1–L2:L3–L4:L5–S1 = 3:3:3

Subgroup analysis revealed biological and anatomical factors modulating osteogenesis (Fig. 7). AFCs from female donors showed a greater calcium deposition trend than male-derived cells, with the triple combination eliciting the strongest response in both sexes. Age stratification indicated enhanced mineralization trend in elderly donors (≥ 60 years) compared with younger patients, with the difference reaching statistical significance under triple treatment (*p* < 0.01). Degeneration-derived AFCs mineralized more than trauma-derived cells across all conditions, and cells from lower lumbar discs (L5–S1) demonstrated a higher osteogenic capacity trend than those from upper lumbar segments (L1–L2). Across all subgroups, the BMP2 + L51P + KMN159 combination consistently yielded the highest mineralization.

### ALP activity levels differed by treatment and donor age

The analysis identified a significant main effect of treatment group (F (6, 41.32) = 6.92, *p* < 0.001), indicating variation in response across the tested conditions. Age group had a significant effect (F (1, 6.87) = 8.53, *p* = 0.023), with higher ALP activity observed in elderly donors (≥ 60 years old). No statistically significant effects were found for sex, disc pathology, spinal level, or passage (Table 3).

**Table 3:** Activity levels differ by treatment and donor age

Predictor	Treatment group	Age group	Sex	Disc pathology	Spinal level	Passage
**p-value**	◍ < 0.001	◍ 0.023	⚪ 0.729	⚪ 0.473	⚪ 0.488	⚪ 0.228
**Predictor**	**Description**			**df**	**F**	**p-value**
Treatment group	Experimental condition (7groups; within-subject factor)			6, 41.32	6.92	< 0.001 ***
Age group	Donors aged < 60 (young) vs. ≥60 years (elderly)			1, 6.87	8.53	0.023 *
Sex	Biological sex (male vs. female)			1, 6.47	0.13	0.729
Disc pathology	Underlying diagnosis (trauma vs. degeneration)			1, 6.21	0.58	0.473
Spinal level	Anatomical level of sample (e.g., L1–2, L3–4)			1, 5.89	0.56	0.488
Passage	Cell passage number (P1–P4)			1, 5.99	1.88	0.228

* Statistically significant (*p* < 0.05); *** Statistically significant (*p* < 0.001)

## Discussion

This study examined the osteogenic potential of combining BMP2, L51P, and KMN159 in human AFCs. The triple treatment consistently enhanced differentiation, as reflected by ALP activity, gene expression, and mineralization. Together, these findings establish a proof-of-concept for a biologically guided spinal fusion strategy that leverages complementary molecular mechanisms. Osteogenic responses were further influenced by donor- and tissue-specific factors, showing the best response in elderly, female patients with some stage of disc degeneration. These outcomes emphasize the importance of personalized biological approaches in spinal fusion.

KMN159 is a difluorolactam EP4 agonist engineered for potent, selective osteoanabolic signaling with favorable chemical stability for local delivery [17]. In vitro, brief exposure of whole bone marrow cultures induces osteoblastic differentiation in an EP4-dependent manner [5]. In vivo, mineralized collagen carriers delivering KMN159 produce dose-dependent regeneration in rat critical-size femoral defects [4] and augment lumbar posterolateral fusion in a preclinical rat model [13]. To our knowledge, this study is the first to apply KMN159 to human AFCs, demonstrating cytocompatibility across 14 days and establishing a rationale for disc-adjacent osteopromotion where controlled, localized bone formation is clinically desirable and where high-dose BMP2 has recognized complications [18].

Recent advances in spinal fusion include combinatorial molecular modulation targeting both BMP antagonists (such as Noggin) and agonists (such as Trb3), which has demonstrated robust bone healing without exogenous BMP2, as well as next-generation biomaterial carriers like supramolecular polymer-collagen microparticle slurries and polyelectrolyte complexes that enable ultra-low BMP2 dosing with high fusion efficacy and reduced adverse effects [19–21]. Synergistic growth factor strategies, such as NELL-1 co-delivery with BMP2, and gene therapy platforms for transient, site-specific BMP2 expression, enhance osteogenesis and fusion rates while minimizing complications, representing a paradigm shift toward safer and more effective biologic spinal fusion approaches [21–23]. The BMP2 variant L51P neutralizes antagonists such as Noggin, Gremlin1, and Chordin, thus releasing BMP2 signaling from negative feedback [24]. In vivo, BMP2 + L51P improved fusion in a rat tail model [6]. In our experiments, groups containing BMP2 showed marked induction of antagonists, but osteogenesis proceeded strongly only when L51P and KMN159 were also present. This suggests that the combination may mitigate inhibitory pressure from upregulated antagonists at the mRNA level. Clinically, this is highly relevant: in fracture repair and cage environments, BMP2 dosing is often escalated to overcome antagonists, at the cost of complications such as ectopic bone formation and inflammation [10, 18]. By attenuating antagonist activity (via L51P) and boosting downstream responsiveness (via EP4 agonism, KMN159), our study demonstrates a path toward BMP2-sparing strategies for fusion. Beyond preserving or enhancing osteoinductive efficacy at lower BMP2 exposure, this approach may also offer safety advantages relative to currently used BMP2 formulations (e.g. InFuse™ฏ, alias InductOs™), which are commonly supplied at 1.5 mg/mL—approximately 15,000-fold higher than the 100 ng/mL BMP2 concentration applied in our in vitro experiments [25].

Fusion outcomes vary by patient age and sex, yet the mechanistic underpinnings remain incompletely understood. While skeletal stem cell studies reported no sex dimorphism [26], our study found that female-derived AFCs mineralized more strongly trend than male-derived cells across most conditions. This may reflect sex-linked epigenetic regulation [27]. Likewise, aging is traditionally associated with reduced regenerative potential; however, in our study, cells from elderly donors exhibited higher mineralization under the triple treatment. This may relate to pro-calcific shifts with senescence and chronic low-grade inflammation (“inflammaging”) [28, 29]. Another critical factor might be that BMP antagonists are present at higher concentrations in healthy discs, typically in younger patients [30]. Clinically, these findings suggest that individual biological characteristics, particularly in elderly or female cohorts, may favor biologically guided fusion strategies, in contrast to the reduced implant fixation observed in these groups. We also observed that degeneration-derived AFCs were more osteogenic than trauma-derived, aligning with the hypothesis that chronic disc degeneration primes cells for osteogenic conversion [31]. In summary, the significant difference observed between degeneration-derived and trauma-derived AFCs suggests that the pathological origin of AFCs represents a dominant modifier of cellular signaling and differentiation behavior, consistent with previous reports showing that degenerative disc cells undergo phenotypic reprogramming, whereas age, sex, and spinal level exert secondary effects [32, 33].

Our data showed that BMP2 alone induced osteogenesis, but the addition of L51P enhanced activity, and the triple treatment with KMN159 achieved the highest ALP activity, bone marker expression, and mineralization. A potential mechanistic hypothesis consistent with these results is that BMP2 initiates osteogenesis, L51P maintains signaling by neutralizing antagonists, and KMN159 amplifies downstream effects via EP4-driven cAMP/PKA and angiogenic programs [34]. In AFCs specifically, mechanotransduction through p38 and SMAD1/5/8 [35] may further synergize with this axis, especially in lower lumbar levels, where we found mineralization was strongest. Thus, the triple combination appears to target three different regulatory checkpoints: induction, release of inhibition, and amplification.

Strengths of our study include the use of primary human AFCs, analysis across a spectrum of donors and disc regions, and multiple osteogenic endpoints. Limitations include the in vitro setting, restricted marker panel, and dependence on osteogenic medium. Whether the combination alone suffices in vivo under native disc conditions remains unknown. Importantly, EP4 signaling intersects with nociceptive PGE2 pathways; while KMN159 has been well tolerated in preclinical bone models [4, 5, 34], its potential to induce pain when applied near disc or nerve tissue must be carefully assessed. Notably, unequal donor sample sizes, particularly the smaller number of trauma-derived IVDs, and overall donor heterogeneity may reduce statistical power and limit the generalizability of subgroup comparisons. Furthermore, functional assessment of BMP signaling, such as SMAD phosphorylation, and protein-level validation of antagonist regulation were not performed.

Future work should extend into disc-relevant co-culture models with nucleus pulposus cells or disc-conditioned media, which have been shown to suppress osteogenesis [9]. Organotypic systems and bioreactors with loading are needed to mimic in vivo conditions [36]. Ultimately, animal models—rat tail fusion for disc space, posterolateral fusion for cage biology—will be essential to validate efficacy, optimize BMP2-sparing doses, and test behavioral pain outcomes. Delivery science will play a key role: mineralized collagen or hydrogel carriers could enable localized, controlled release, reduce peak exposure and minimize nociceptive spillover.

## Conclusion

The combination of BMP2, L51P, and KMN159 enhanced osteogenic differentiation of human AFCs in vitro, with particularly strong effects in cells from elderly, female, and degeneration-derived discs. This might lead to successful strategies to induce or enhance fusion in the IVD space.

## Data Availability

All the data used in this research is available on request from the first and corresponding author.

## References

[1] Chanchairujira K, Chung CB, Kim JY, Papakonstantinou O, Lee MH, Clopton P, Resnick D (2004) Intervertebral disk calcification of the spine in an elderly population: radiographic prevalence, location, and distribution and correlation with spinal degeneration. Radiology 230:499–503. 10.1148/radiol.2302011842 14752191

[2] Saremi A, Goyal KK, Benzel EC, Orr RD (2024) Evolution of lumbar degenerative spondylolisthesis with key radiographic features. Spine J 24. 10.1016/j.spinee.2024.01.001 38199449

[3] Veronesi F, Sartori M, Griffoni C, Valacco M, Tedesco G, Davassi PF, Gasbarrini A, Fini M, Barbanti Brodano G (2022) Complications in Spinal Fusion Surgery: A Systematic Review of Clinically Used Cages. J Clin Med 11. 10.3390/jcm11216279 PMC9659217 36362508

[4] Vater C, Mehnert E, Bretschneider H, Bolte J, Findeisen L, Matuszewski L-M, Zwingenberger S (2021) Dose-Dependent Effects of a Novel Selective EP4 Prostaglandin Receptor Agonist on Treatment of Critical Size Femoral Bone Defects in a Rat Model. Biomedicines 9. 10.3390/biomedicines9111712 PMC8615441 34829941

[5] Owen TA, Patel C, Wei S, Ho CS, Birmingham K, Sanchez S, Chung N, Cahill A, O’Malley JP, Barrett SD et al (2020) KMN-159, a novel EP4 receptor selective agonist, stimulates osteoblastic differentiation in cultured whole rat bone marrow. Gene 748:144668. 10.1016/j.gene.2020.144668 PMC7416767 32334025

[6] Gantenbein B, Oswald KAC, Erbach GF, Croft AS, Bermudez-Lekerika P, Strunz F, Bigdon SF, Albers CE (2024) The bone morphogenetic protein 2 analogue L51P enhances spinal fusion in combination with BMP2 in an in vivo rat tail model. Acta Biomater 177:148–156. 10.1016/j.actbio.2024.01.039 38325708

[7] Xu Y, Yang Y, Hua Z, Li S, Yang Z, Liu Q, Fu G, Ji P, Wu Q (2021) BMP2 immune complexes promote new bone formation by facilitating the direct contact between osteoclasts and osteoblasts. Biomaterials 275:120890. 10.1016/j.biomaterials.2021.120890 34130144

[8] Panos JA, Coenen MJ, Nagelli CV, McGlinch EB, Atasoy-Zeybek A, De Padilla CL, Coghlan RF, Johnstone B, Ferreira E, Porter RM et al (2023) IL-1Ra gene transfer potentiates BMP2-mediated bone healing by redirecting osteogenesis toward endochondral ossification. Mol Ther 31:420–434. 10.1016/j.ymthe.2022.10.007 PMC9931547 36245128

[9] Chen S, Croft AS, Bigdon S, Albers CE, Li Z, Gantenbein B (2024) Conditioned Medium of Intervertebral Disc Cells Inhibits Osteo-Genesis on Autologous Bone-Marrow-Derived Mesenchymal Stromal Cells and Osteoblasts. Biomedicines 12. 10.3390/biomedicines12020376 PMC10886592 38397978

[10] Hsu M-N, Yu F-J, Chang Y-H, Huang K-L, Pham NN, Truong VA, Lin M-W, Kieu Nguyen NT, Hwang S-M, Hu Y-C (2020) CRISPR interference-mediated noggin knockdown promotes BMP2-induced osteogenesis and calvarial bone healing. Biomaterials 252:120094. 10.1016/j.biomaterials.2020.120094 32422495

[11] Albers CE, Hofstetter W, Sebald H-J, Sebald W, Siebenrock KA, Klenke FM (2012) L51P - A BMP2 variant with osteoinductive activity via inhibition of Noggin. Bone 51:401–406. 10.1016/j.bone.2012.06.020 22750402

[12] Chernysheva M, Ruchko E, Eremeev A, Optimizing (2025) rhBMP-2 Therapy for Bone Regeneration: From Safety Concerns to Biomaterial-Guided Delivery Systems. Int J Mol Sci 26. 10.3390/ijms262110723 PMC12609753 41226759

[13] Findeisen L, Tian X, Vater C, Raina DB, Kern H, Bolte J, Straßburger L, Matuszewski L-M, Modler N, Gottwald R et al (2025) Exploring an innovative augmentation strategy in spinal fusion: A novel selective prostaglandin EP4 receptor agonist as a potential osteopromotive factor to enhance lumbar posterolateral fusion. Biomaterials 320:123278. 10.1016/j.biomaterials.2025.123278 40132358

[14] Hiyama A, Yokoyama K, Nukaga T, Sakai D, Mochida J (2015) Response to tumor necrosis factor-α mediated inflammation involving activation of prostaglandin E2 and Wnt signaling in nucleus pulposus cells. J Orthop Res 33:1756–1768. 10.1002/jor.22959 26123748

[15] May RD, Frauchiger DA, Albers CE, Hofstetter W, Gantenbein B (2020) Exogenous Stimulation of Human Intervertebral Disc Cells in 3-Dimensional Alginate Bead Culture With BMP2 and L51P: Cytocompatibility and Effects on Cell Phenotype. Neurospine 17:77–87. 10.14245/ns.2040002.001 PMC7136110 32252157

[16] Livak KJ, Schmittgen TD (2001) Analysis of Relative Gene Expression Data Using Real-Time Quantitative PCR and the 2 – ∆∆CT Method. Methods 25:402–408. 10.1006/meth.2001.1262 11846609

[17] Barrett SD, Holt MC, Kramer JB, Germain B, Ho CS, Ciske FL, Kornilov A, Colombo JM, Uzieblo A, O’Malley JP et al (2019) Difluoromethylene at the γ-Lactam α-Position Improves 11-Deoxy-8-aza-PGE1 Series EP4 Receptor Binding and Activity: 11-Deoxy-10,10-difluoro-8-aza-PGE1 Analog (KMN-159) as a Potent EP4 Agonist. J Med Chem 62:4731–4741. 10.1021/acs.jmedchem.9b00336 30964292

[18] Tannoury CA, An HS (2014) Complications with the use of bone morphogenetic protein 2 (BMP-2) in spine surgery. Spine J 14:552–559. 10.1016/j.spinee.2013.08.060 24412416

[19] McClendon MT, Ji W, Greene AC, Sai H, Sangji MH, Sather NA, Chen CH, Lee SS, Katchko K, Jeong SS et al (2023) A supramolecular polymer-collagen microparticle slurry for bone regeneration with minimal growth factor. Biomaterials 302:122357. 10.1016/j.biomaterials.2023.122357 PMC10897953 37879188

[20] Fan J, Zhang X, Kang M, Lee C-S, Kim L, Hadaya D, Aghaloo TL, Lee M (2023) Complementary modulation of BMP signaling improves bone healing efficiency. Biomaterials 302:122335. 10.1016/j.biomaterials.2023.122335 PMC10805245 37748419

[21] Liu L, Lam WMR, Naidu M, Yang Z, Wang M, Ren X, Hu T, Kumarsing R, Ting K, Goh JC-H et al (2019) Synergistic Effect of NELL-1 and an Ultra-Low Dose of BMP-2 on Spinal Fusion. Tissue Eng Part A 25:1677–1689. 10.1089/ten.TEA.2019.0124 31337284

[22] De la Vega RE, Atasoy-Zeybek A, Panos JA, Van Griensven M, Evans CH, Balmayor ER (2021) Gene therapy for bone healing: lessons learned and new approaches. Transl Res 236. 10.1016/j.trsl.2021.04.009 PMC8976879 33964474

[23] Cottrill E, Pennington Z, Sattah N, Jing C, Salven D, Johnson E, Downey M, Varghese S, Rocos B, Richardson W (2024) Gene Therapy and Spinal Fusion: Systematic Review and Meta-Analysis of the Available Data. World Neurosurg 186. 10.1016/j.wneu.2024.03.174 38583566

[24] Khattab HM, Ono M, Sonoyama W, Oida Y, Shinkawa S, Yoshioka Y, Maekawa K, Tabata Y, Sugama K, Sebald W et al (2014) The BMP2 antagonist inhibitor L51P enhances the osteogenic potential of BMP2 by simultaneous and delayed synergism. Bone 69:165–173. 10.1016/j.bone.2014.09.011 25240457

[25] Karipidou N, Gorley JPM, Katrilaka C, Manglaris C, Tzavellas AN, Pitou M, Cheva A, Michailidis N, Tsiridis EE, Choli-Papadopoulou T et al (2025) A Critical Review of Commercial Collagen-Based Scaffolds in Bone Regeneration: Functional Properties and Clinical Evidence from Infuse^®^ Bone Graft. J Funct Biomaterials 16. 10.3390/jfb16090313 PMC12470532 41003384

[26] Niemann T, Joneleit J, Storm J, Nacke T, Wähnert D, Kaltschmidt C, Vordemvenne T, Kaltschmidt B (2023) Analyzing Sex-Specific Dimorphism in Human Skeletal Stem Cells. Cells 12. 10.3390/cells12232683 PMC10705359 38067111

[27] Grant OA, Wang Y, Kumari M, Zabet NR, Schalkwyk L (2022) Characterising sex differences of autosomal DNA methylation in whole blood using the Illumina EPIC array. Clin Epigenetics 14:62. 10.1186/s13148-022-01279-7 PMC9107695 35568878

[28] Kushioka J, Chow SK-H, Toya M, Tsubosaka M, Shen H, Gao Q, Li X, Zhang N, Goodman SB (2023) Bone regeneration in inflammation with aging and cell-based immunomodulatory therapy. Inflamm Regen 43:29. 10.1186/s41232-023-00279-1 PMC10210469 37231450

[29] Yang X, Wang Y, Rovella V, Candi E, Jia W, Bernassola F, Bove P, Piacentini M, Scimeca M, Sica G et al (2023) Aged mesenchymal stem cells and inflammation: from pathology to potential therapeutic strategies. Biol Direct 18:40. 10.1186/s13062-023-00394-6 PMC10353240 37464416

[30] Chen S, Bigdon SF, Häckel S, Li Z, Albers CE, Gantenbein B (2025) Noggin expression in human nucleus pulposus cells: A comparative study of degenerative and traumatic intervertebral discs. Adv Orthop 1:55–62. 10.1016/j.advop.2025.04.001

[31] Novais EJ, Narayanan R, Canseco JA, van de Wetering K, Kepler CK, Hilibrand AS, Vaccaro AR, Risbud MV (2024) A new perspective on intervertebral disc calcification-from bench to bedside. Bone Res 12:3. 10.1038/s41413-023-00307-3 PMC10803356 38253615

[32] Rodrigues-Pinto R, Richardson SM, Hoyland JA (2014) An understanding of intervertebral disc development, maturation and cell phenotype provides clues to direct cell-based tissue regeneration therapies for disc degeneration. Eur Spine J 23:1803–1814. 10.1007/s00586-014-3305-z 24777668

[33] Hunter DJ, Schofield D, Callander E (2014) The individual and socioeconomic impact of osteoarthritis. Nat Rev Rheumatol 10:437–441. 10.1038/nrrheum.2014.44 24662640

[34] Ukon Y, Nishida M, Yamamori N, Takeyama K, Sakamoto K, Takenaka S, Makino T, Fujimori T, Sakai Y, Kanie Y et al (2022) Prostaglandin EP4 Selective Agonist AKDS001 Enhances New Bone Formation by Minimodeling in a Rat Heterotopic Xenograft Model of Human Bone. Front Bioeng Biotechnol 10:845716. 10.3389/fbioe.2022.845716 PMC8968459 35372320

[35] Chen C-N, Chang H-I, Yen C-K, Liu W-L, Huang K-Y (2022) Mechanical Stretch Induced Osteogenesis on Human Annulus Fibrosus Cells through Upregulation of BMP-2/6 Heterodimer and Activation of P38 and SMAD1/5/8 Signaling Pathways. Cells 11. 10.3390/cells11162600 PMC9406707 36010676

[36] Gantenbein B, Illien-Jünger S, Chan SCW, Walser J, Haglund L, Ferguson SJ, Iatridis JC, Grad S (2015) Organ culture bioreactors–platforms to study human intervertebral disc degeneration and regenerative therapy. Curr Stem Cell Res Therapy 10:339–352. 10.2174/1574888x10666150312102948 PMC4437861 25764196

